# Incorporation of Exogenous RGD Peptide and Inter-Species Blending as Strategies for Enhancing Human Corneal Limbal Epithelial Cell Growth on *Bombyx mori* Silk Fibroin Membranes

**DOI:** 10.3390/jfb4020074

**Published:** 2013-05-17

**Authors:** Laura J. Bray, Shuko Suzuki, Damien G. Harkin, Traian V. Chirila

**Affiliations:** 1 Queensland Eye Institute, South Brisbane, Queensland 4101, Australia; E-Mails: laura.bray@qut.edu.au (L.J.B.); shuko.suzuki@qei.org.au (S.S.); d.harkin@qut.edu.au (D.G.H.); 2 Faculty of Health, Queensland University of Technology, Brisbane, Queensland 4001, Australia; 3 Institute of Health & Biomedical Innovation, Kelvin Grove, Queensland 4059, Australia; 4 Faculty of Science and Engineering, Queensland University of Technology, Brisbane, Queensland 4001, Australia; 5 Faculty of Health Sciences, University of Queensland, Herston, Queensland 4006, Australia; 6 Australian Institute of Bioengineering and Nanotechnology, University of Queensland, St. Lucia, Queensland 4072, Australia; 7 Faculty of Science, University of Western Australia, Crawley, Western Australia 6009, Australia

**Keywords:** silk fibroin, corneal limbal epithelial cells, RGD peptide, fibroin blends, cell adhesion

## Abstract

While fibroin isolated from the cocoons of domesticated silkworm *Bombyx mori* supports growth of human corneal limbal epithelial (HLE) cells, the mechanism of cell attachment remains unclear. In the present study we sought to enhance the attachment of HLE cells to membranes of *Bombyx mori* silk fibroin (BMSF) through surface functionalization with an arginine-glycine-aspartic acid (RGD)-containing peptide. Moreover, we have examined the response of HLE cells to BMSF when blended with the fibroin produced by a wild silkworm, *Antheraea pernyi*, which is known to contain RGD sequences within its primary structure. A procedure to isolate *A. pernyi* silk fibroin (APSF) from the cocoons was established, and blends of the two fibroins were prepared at five different BMSF/APSF ratios. In another experiment, BMSF surface was modified by binding chemically the GRGDSPC peptide using a water-soluble carbodiimide. Primary HLE were grown in the absence of serum on membranes made of BMSF, APSF, and their blends, as well as on RGD-modified BMSF. There was no statistically significant enhancing effect on the cell attachment due to the RGD presence. This suggests that the adhesion through RGD ligands may have a complex mechanism, and the investigated strategies are of limited value unless the factors contributing to this mechanism become better known.

## 1. Introduction

Silk proteins have been introduced as biomaterials in the early 1990s through Minoura’s seminal papers [1,2,3]. The two principal constitutive proteins (fibroin and sericin) of the silk threads produced by the larvae of certain silkmoths in class *Insecta* have been investigated extensively for biomaterials and tissue engineering applications [4,5,6,7,8,9,10,11,12,13,14,15], although apparently little or no successful usage in a clinical setting has been reported so far [16].

In most applications, a prerequisite for any biomaterial is its ability to function effectively as a substratum for the attachment and growth of a large variety of cells that are specific to the human tissue with which the biomaterial must come in direct contact and interact. The silk fibroin isolated from the cocoons produced by the larvae of domesticated silkmoth *Bombyx mori* (family *Bombycidae*), (BMSF), has been by far the most investigated silk substratum for cells [2,3,5,17,18,19,20,21]. Realistically, however, the cell attachment to the BMSF surface can be described at best as satisfactory, if not even weak [22]. Considering the essential role played in cell adhesion process by the integrin binding sites located on the cell surface, the mechanism of cell attachment to silk fibroins is debatable and worth to investigate. It is known that the integrin receptors interact specifically with certain peptide domains (ligands) present in the extracellular matrix components, and that these integrin-binding domains are ultimately responsible for the adhesion and survival of anchorage-dependent cells. A typical ligand peptide motif is the arginine-glycine-aspartic acid (RGD) sequence found in fibronectin. When such motifs are present on a substratum, and are also sterically exposed, they can promote the cells’ attachment, followed by spreading, proliferation and differentiation. However, BMSF does not contain RGD or any other known ligand peptide motif [2,23,24,25]. On the other hand, the fibroins isolated from the silk produced by the larvae of wild silkmoths in the family *Saturniidae*, genus *Antheraea*, such as *A. pernyi*, *A. mylitta* and *A. yamamai*, which do not feed on mulberry leaves, contain RGD domains in their structure [2,26,27,28,29,30,31,32], and thus are perceived to be more suitable as substrata for cells. The absence in BMSF of adhesion peptide domains makes it rather difficult to explain its cell-adhesive properties, which have been indeed proved suitable for growing a variety of cells. It was suggested [2] that the large proportion of arginine present in fibroin’s composition may contribute to this process. An alternative suggestion [3,33] has been that cell adhesion on BMSF could be the result of electrostatic interactions between the negatively charged cell surface (glycocalyx) and the positively charged amine residues in the fibroin. If so, the process must be regarded as non-specific. Yet, searching for specific interactions in this process, Tsubouchi *et al*. [34] isolated (by enzymatic digestion) two peptides located near the N-terminal region of the heavy chain of BMSF, namely a decapeptide (VITTDSDGNE) and an octapeptide (NINDFDED), which were assumed to be hitherto unknown adhesion ligands. There has been no confirmation so far of this assumption. Likely, the cell-adhesive properties of BMSF may be a result of a favorable combination of non-specific interactions based on surface characteristics (charge, wettability and topography).

A strategy for enhancing the cell adhesion to BMSF has been the incorporation of ligand peptide motifs (usually RGD sequences). Preferred techniques include chemical functionalization of BMSF with adhesion motifs by binding them covalently to the protein [9,35,36,37], “genetical” functionalization with interfused peptide motifs through recombinant techniques [38,39,40,41], or blending BMSF with isolated components of the extracellular matrix, such as elastin [42,43]. Other strategies, based on the possibility to enhance non-specific interactions, included [22] the manipulation of surface charge, wettability, or topography of the BMSF substrata.

In this study, we attempted to enhance the cell-adhesivity of BMSF by blending it with *A. pernyi* silk fibroin (APSF), which is known to contain RGD domains. Membranes of various compositions were made and used as substrata for growing cells. The effect of increasing amounts of APSF in blends on cell attachment and growth was also investigated. In addition, RGD adhesion domains were incorporated on the surface of BMSF membranes by chemical functionalization, and the growth of same cells was comparatively assessed. Primary cultures of human corneal limbal epithelial (HLE) cells have been used as our model system owing to our interest in the *ex vivo* cultivation and transplantation of these cells for the treatment of severe eye diseases and reconstruction of ocular surface. 

## 2. Materials and Methods

### 2.1. Materials

The *B. mori* silkworm cocoons were supplied by Tajima Shoji Co Ltd (Yokohama, Japan), and the *A. pernyi* silkworm cocoons by the Lepidoptera Breeders Association (Sleaford, UK). All chemicals were purchased from Sigma-Aldrich (St. Louis, MO, USA), with the exceptions noted here. The GRGDSPC (Gly-Arg-Gly-Asp-Ser-Pro-Cys) peptide (purity 98%) was purchased from GL Biochem Ltd. (Shanghai, China). Water of high purity (Milli-Q or equivalent) was used in all experiments. Minisart® filters (20 μm) and Minisart®-GF pre-filters (80 μm) were supplied by Sartorius Stedim Biotech (Göttingen, Germany). The dialysis cassettes (Slide-A-Lyzer®) (MWCO 3.5 kDa) were purchased from Thermo Scientific (Rockford, IL, USA), and the dialysis tubing with MWCO 12.4 kDa from Sigma-Aldrich.

All cell culture reagents were purchased from Life Technologies Inc. (Mulgrave, Australia), with the following exceptions. Foetal bovine serum (FBS) was purchased from Thermo Scientific (Scoresby, Australia). 3,3,5-Triiodo-L-thyronine sodium salt (T_3_), adenine, transferrin, hydrocortisone, insulin, tris(hydroxymethyl)aminomethane, and EDTA were all purchased from Sigma-Aldrich (St. Louis, MO, USA). Isoproteranol was purchased from Merck (Kilsyth, Australia). Sterile ethanol 70% for sterilization was supplied by ORION Laboratories (Perth, Australia).

### 2.2. Preparation of Fibroin Solutions

A solution of regenerated BMSF was prepared according to a protocol previously reported [17], leading in the particular batch for this study to a concentration of 3% BMSF (by gravimetric analysis).

In order to obtain the solution of regenerated APSF, the cocoons were first dried, cut into approximately 1 × 1 cm pieces, weighed, and then placed in a 1-L beaker with a boiling solution of sodium carbonate containing 0.85 g salt for 1 g of cocoon material. During 1 h of boiling, the fibres were occasionally pulled gently apart using tweezers. After boiling, the supernatant was discarded, the fibrous material squeezed to remove the excess liquid, and then treated for 20 min, three times in succession, in 1 L of warm (60 °C–70 °C) water, followed by drying in a ventilated fume hood for at least 12 h. The dry fibrous mass was mixed with neat calcium nitrate tetrahydrate (20 times weight excess to the amount of fibres) at 105 °C and kept for 5 h on an oil bath while stirring very slowly. The resulting solution was aspirated in a syringe and injected into pre-treated (4-h soaking, with 5 water exchanges) dialysis tubing (MWCO 12.4 kDa), which was then placed into a 1-L beaker with chilled water (4 °C) and kept in a refrigerator. Water was exchanged for fresh pre-chilled water 6 times at increasing intervals over 3 days of dialysis. The resulting fibroin solution was removed carefully from the dialysis tubing and filtered successively through 0.8 and 0.2 µm filters into a dialysis cassette (MWCO 3.5 kDa) in pre-chilled (4 °C) 30% wt/vol aqueous solution of poly(ethylene glycol) (MW 10 kDa), and left to be dialysed for about 10 h. The solution collected in this particular batch from the dialysis cassette contained 1.4% APSF (by gravimetric analysis).

### 2.3. Preparation of Fibroin Membranes

The BMSF and APSF membranes were cast from their respective solutions produced as described above. For making the blended membranes, the two fibroin solutions were mixed together to provide mixtures with the following compositions (BMSF/APSF, in % wt/wt): 90/10, 70/30, 50/50, 30/70 and 10/90. Prior to casting, all solutions were allowed to homogenize at 4 °C for 3 h in a refrigerator. The membranes for cell attachment studies, around 10 μm in thickness, were cast directly in the wells of 24-well tissue culture plates, starting with 265 μL solution in each well. Prior to the water annealing, the membranes rich in BMSF (≤50% APSF) were placed in a fan-driven oven and kept for 12 h at room temperature; the membranes with higher APSF contents had to be dried at 4 °C (refrigerator) for 2 weeks, in order to avoid premature phase separation (cloudiness). After drying, the coated plates were placed in a vacuum enclosure, where they were annealed at –80 kPa and room temperature in the presence of water (in a beaker). The annealing duration for the membranes rich in BMSF (70%, 90% and 100%) was 6 h, while for the others was 24 h, which assured their complete insolubility in water. 

### 2.4. Chemical Functionalization of BMSF with an RGD Peptide

The BMSF membranes were cast and processed in the wells of 24-well tissue culture plates. Annealed and dry BMSF membranes were functionalized with an RGD-containing peptide (GRGDSPC) following a slightly modified version of a reported method [44]. In brief, the carboxyl groups in BMSF were activated with 0.5 mg/mL *N*-(3-dimethylaminopropyl)-*N'*-ethylcarbodiimide hydrochloride (EDC) and 0.7 mg/mL *N*-hydroxysuccinimide (NHS) in 0.1 M MES buffer for 45 min at room temperature. (The MES buffer was prepared by dissolving 4-morpholineethanesulfonic acid hydrate in an aqueous solution of 0.3 M NaCl and adjusting to pH 6.5). The GRGDSPC solution in MES buffer, containing either 0.1 mg/mL or 0.5 mg/mL peptide, was added to the wells and left for 3 h at room temperature. The membranes in wells were then copiously rinsed with buffer and water, and the plates were then placed in a fan-driven oven and kept for 6 h at room temperature.

### 2.5. X-ray Photoelectron Spectroscopy (XPS)

The presence of the RGD domains on the surface of BMSF after chemical modification was assessed by XPS, based on detecting the presence of sulfur (in the cysteine structural units) as a marker for the entire sequence, a procedure that has been previously used [45]. The membranes were removed from wells and additionally dried in a fan-driven oven for 6 h at ambient temperature. Three samples of each modified BMSF, respectively with 0.1 mg/mL and with 0.5 mg/mL, and a non-treated BMSF membrane (as a control) were analyzed in an Axis Ultra XPS instrument (Kratos Analytical, Manchester, UK). The source of the incident X-ray radiation was the monochromatic Al Kα (1486.6 eV) operating at 150 W (15 kV, 10 mA). The overall information depth was about 10 nm. Survey scans were taken at the analyzer pass energy of 160 eV and 0.5 eV/step. Binding energies were calibrated by setting the signal of aliphatic C 1*s* at 285 eV. The atom percentages of sulfur were calculated for each sample in triplicate and averaged. 

### 2.6. Contact Angle Analysis

For this analysis, the membranes of BMSF, APSF and their 50/50 (wt/wt) blend were cast and annealed directly onto microscope glass slides that were not removed prior to measurements. Contact angles were measured after placing a water droplet onto the dry annealed membranes. A water droplet of about 5 µL was applied onto the membrane surface, and photographs were taken with a Sanyo VCB-3512 T CCD camera at an interval of 5 s after the droplet was dispensed. The resulting contact angle was measured in a goniometer (FTÅ200, First Ten Ångstroms, Inc., Portsmouth, NH, USA) using the FTÅ Drop Shape Analysis Software Version 2.0 (2002). The results reported are the average values of 16 measurements for each membrane.

### 2.7. Establishment of Primary HLE Cultures

Ocular tissue was collected as either corneoscleral rims or corneoscleral caps, both provided by the Queensland Eye Bank (QEB), Brisbane, Australia. The tissue was washed three times with PBS for 10 min, sectioned into quarters, and subsequently incubated with 0.25% dispase at 37 °C for 1 h. The dissociated HLE sheets from the corneoscleral quadrants were collected, pooled, centrifuged at 300 *g* for 5 min and finally re-suspended in 0.25% trypsin in 0.2 g/L EDTA for 5 min. Cells were washed with serum-containing medium, centrifuged at 300 *g* for 5 min and re-suspended in serum-supplemented culture medium. HLE cultures were propagated in the presence of irradiated 3T3 murine fibroblast feeder cells as described previously [46]. Passage 1 cultures were further employed for the cell attachment assays. 

### 2.8. Cell Attachment Assay

Cell attachment assays were performed as per manufacturer’s instructions for the Quant-iT PicoGreen dsDNA Assay (Life Technologies Inc., Carlsbad, CA, USA). Briefly, 2 × 10^4^ HLE cells/cm^2^ were seeded onto BMSF, APSF, their blends, or RGD-functionalised BMSF coated in the wells of a 24-well plate. Prior to seeding, the coated wells were sterilized with 70% ethanol, followed by extensive rinsing with PBS. Cells were incubated for 4 h in serum-free medium, washed twice in PBS, and then 1 mL of 0.1% Triton-X100 was added. The plates were then incubated at room temperature for 1 h. Each well was then triturated and the supernatant was transferred into individual 2 mL Eppendorf tubes. The tubes were centrifuged at maximum speed for 5 min. To set up the PicoGreen analysis, the samples were aliquoted at 25 µL each into a 96-well plate with 75 µL of 10 mM Tris-HCl in 1 mM EDTA buffer (TE buffer). PicoGreen dye was then added at 1:200 dilution in TE buffer to each well in 100 µL portions. The plate was read on fluorescent microplate reader (FLUOstar OPTIMA, BMG Labtech Pty Ltd., Mornington, Australia). DNA content, calculated and plotted in ng/mL, relates directly to the cell number. These experiments were conducted a minimum of 5 times for each series of experiments using cells obtained from a different tissue donor on each occasion.

### 2.9. Statistical Analysis

For all results that needed to be statistically processed, the one-way analysis of variance (ANOVA) in conjunction with Tukey-Kramer multiple comparisons test was employed, using the GraphPad InStat® Version 3.10, or GraphPad Prism® Version 6.0.

## 3. Results

### 3.1. Characterization of BMSF/APSF Blends

The blend membranes displayed similar handling properties to BMSF alone, however an increasing content of APSF made the membranes more brittle and prone to breaking, which was substantiated quantitatively in a separate study [47].

The values measured for the contact angles onto the surface of the membranes made of BMSF, APSF and their equivalent blend (50/50 wt/wt) were, respectively, 48.4 ± 1.2º, 62.4 ± 1.0 and 50.0 ± 0.07º (as mean values ± SEM for *n* = 16). These values join the values reported by others [35,48,49,50,51,52] in a range that is notoriously wide, perhaps reflecting the method variability in preparing the membranes. We are not aware of any value having been reported for APSF. Our analysis revealed a trend towards decreased hydrophilicity with increasing APSF content. The measured values put all three membranes in the category of materials that promote cell attachment [53].

### 3.2. Functionalization of BMSF with GRGDSPC

Successful binding of the RGD-containing peptide to the surface of BMSF membranes was confirmed by XPS analysis. A signal corresponding to sulphur was observed as a S 2*p* composite peak (an unresolved doublet) in the spectra of modified BMSF at binding energies of 160 to 166 eV. The atom-percentages of S were 0.04 ± 0.02 at.-% in the sample control (unmodified BMSF), 0.09 ± 0.00 at.-% in the sample modified with 0.1 mg/mL GRGDSPC, and 0.12 ± 0.02 at.-% in the sample modified with 0.5 mg/mL GRGDSPC in the reaction medium, as mean values ± SEM (*n* = 3). This region can be assigned to the sulphide bond and has been used as a signature for cysteine-containing peptides immobilized to synthetic polymers [54,55]. The weak intensity of the S 2*p* signal may be related to a low penetration of the reaction activating agents [55,56], which restricts the presence of the bound peptide within an uppermost layer much thinner than the depth probed by the XPS (about 10 nm). As most of the XPS-probed substratum did not react with the peptide, it does not contain additional sulphur, and the resulting numerical value is diminished due to a greater background mass. 

### 3.3. Response of HLE Cells to BMSF, APSF and Blends

**Figure 1:**
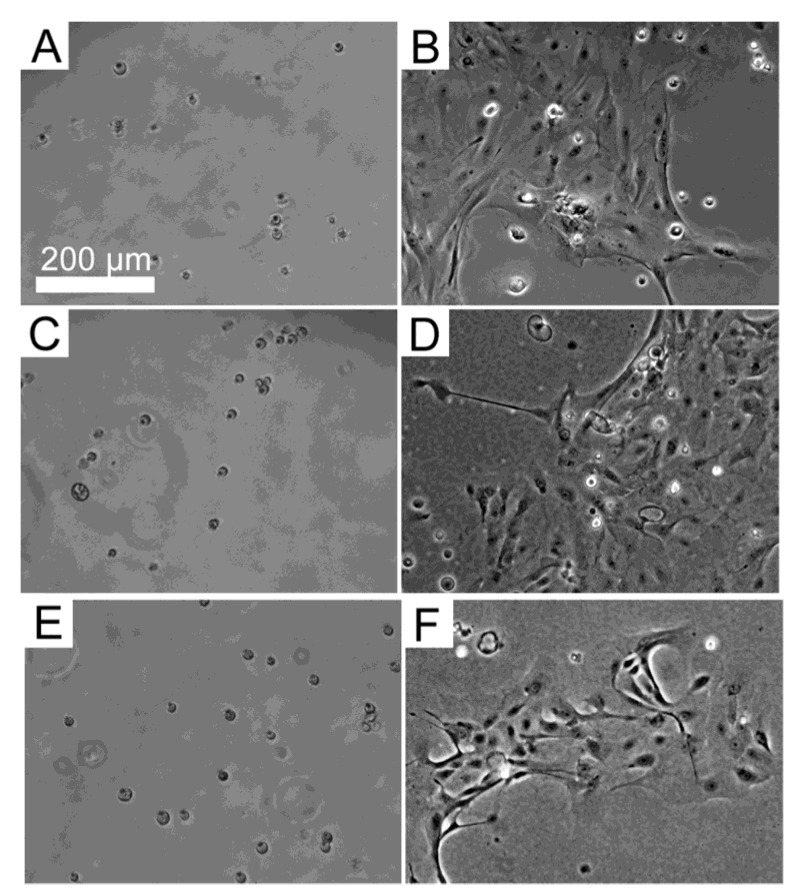
Primary human corneal limbal epithelial (HLE) cells grown on (**A,B**) BMSF; (**C,D**) APSF; and (**E,F**) their 50/50 BMSF/APSF blend substrata. Panels A, C and E show the attachment of cells after 4 h. Panels B, D and F show their growth after 48 h. The scale bar is the same for all panels.

**Figure 2:**
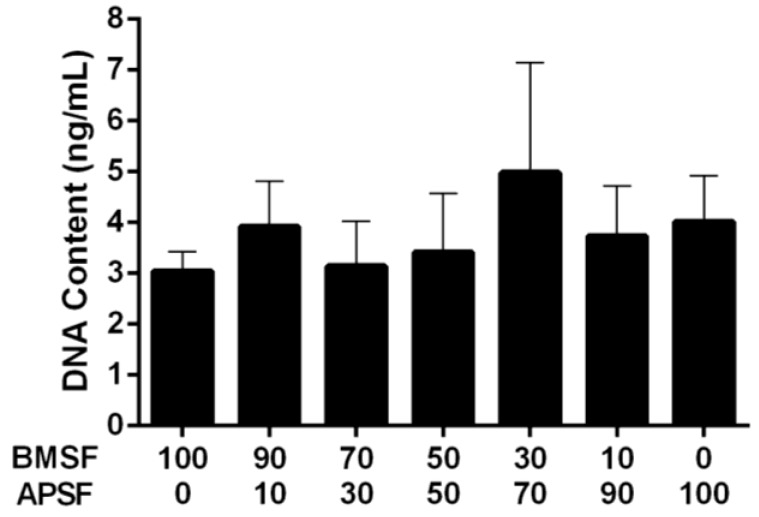
Quantitative comparison of the HLE cell attachment to BMSF, APSF and their blends, when seeded in the absence of serum. Bars represent the mean value ± SEM for the total number of viable cells assessed after 4 h by the DNA content *via* PicoGreen assay.

Primary cultures of HLE cells established from donor human eye tissue displayed cobblestone morphology and achieved confluence within approximately 14 days. When subsequently passaged and re-suspended in serum-free culture medium, the HLE cells attached to the substrata, and then maintained their morphology (Figure 1). The level of attachment to the BMSF/APSF coatings was variable (Figure 2). Low numbers of cells were typically seen on 100% BMSF, while a higher number was recorded on the BMSF 30/APSF 70 membrane. The cells on all other coatings, including both the blends and the 100% APSF, displayed varying results, with no consistent pattern as a function of the composition of blends. The differences in cell attachment were not statistically significant.

### 3.4. Response of HLE Cells to RGD-Functionalized BMSF

**Figure 3:**
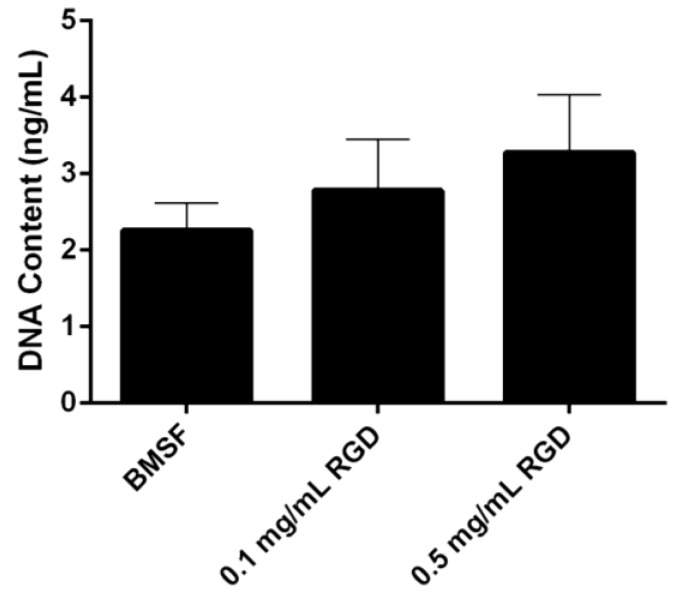
Quantitative comparison of the HLE cell attachment to BMSF and BMSF modified with an RGD-containing peptide. Bars represent the mean value ± SEM for the total number of viable cells assessed after 4 h by the DNA content *via* PicoGreen assay.

A dose-dependent increase was observed in the level of HLE cells attached to the BMSF membranes modified with an RGD peptide (Figure 3). This notwithstanding, the results were not statistically significant.

## 4. Discussion

The regeneration (*i.e.*, degumming, solubilization, and casting) of APSF is more difficult than that of BMSF due to its stronger resistance to chemical agents, which is caused by extended β-sheet regions and hydrogen bonding [57]. Indeed, we had difficulties in employing some of the published protocols [57,58,59,60], and concluded that the source of the *A. pernyi* silk cocoons might be a contributing factor. Accordingly, we established a protocol [61] that was suitable for the cocoons used in this study.

Amongst the fibroins isolated from the so-called “non-mulberry” silks produced by silkworms of the genus *Antheraea*, known also as “tussah” or “tasar” silks, which all contain RGD domains in their primary structure, the one isolated from *A. mylitta* (“tropical” or Indian tussah) has been extensively investigated as a substratum for cells by Kundu’s group [31,32,62,63,64,65]. It was found [64] that, when grown on *A. mylitta* silk fibroin membranes in the presence of serum, the cells developed actin filaments and junctions with the substratum to a higher level than with tissue culture plastic or BMSF. These processes were located not only at the periphery of cells, but also distributed throughout. The formation of focal adhesions, mature actin fibres and, ultimately, tubulin microtubes have been attributed to the presence of RGD domains in the fibroin. 

Attachment and growth of cells on APSF has been investigated to a lesser extent [2,66,67,68]. Minoura *et al*. [2] demonstrated superior cell attachment and growth on APSF when compared to BMSF or collagen substrata, using a L-929 cell line, in the presence of serum. The ratios between the cell numbers on test substrata and tissue culture plastic after 17 h and after 50 h, arbitrarily defined as “attachment ratio” and, respectively, “growth ratio”, were found to be approximately 1.5 times higher on APSF. A favorable effect of APSF on L-929 cells’ growth in blends with BMSF has been also reported [66] using porous films prepared by freeze-drying and then chemically crosslinked. The cells proliferated in the highest number on APSF, and there was also a dose-dependent increase with the APSF content. (Other details and quantitative data are not available in this reference that can be accessed in English only as a summary.) In another study [67], the growth of neurons isolated from the rat brain has been investigated on both films and nanofibres of BMSF, APSF, their 50/50 blend and poly(L-lysine) (as a positive control) in serum-free conditions. The cells attached to all substrata, but those on APSF displayed dendritic morphology while those on BMSF and the blend showed a simple morphology. A quantitative analysis of the dendritic processes confirmed the better performance of APSF as a substratum for neurons. In a recent study [68], the growth and metabolism of human microvascular endothelial cells were compared when cells were seeded in the presence of serum on “industrially degummed”, commercially available BM and AP (therefore presumed fibroins), and on a spider silk (*Nephila edulis*). There were many aspects investigated in this study, but for our discussion it is important to note that cell growth and metabolism were significantly diminished on APSF as compared to the other substrata. In fact, after 7 days in culture, APSF induced complete cell death. Additional assays indicated that the commercial APSF is cytotoxic/cytostatic, due to the interaction of a component of the silk surface with a component of the serum, the former being located between the fibroin fibres and the sericin layer. This suggested an incomplete degumming process, and indeed if the commercial APSF was additionally treated with trypsin to clean its surface, the substratum ceased to be toxic. There was no finding of an enhanced cell growth due to APSF.

In our present study, neither the grafting of RGD adhesion domains onto the surface of BMSF, nor its blending with the RGD-containing APSF were found to have a significant effect on the attachment of HLE cells. There is a definite trend towards enhanced (rather than reduced) cell attachment, as clearly seen in Figure 2, Figure 3, however when subjected to the standards of statistical rigor, the data loose their significance and no valid prediction can be made. Other investigators reported [44] improved growth of corneal stromal cells on RGD-functionalized BMSF. This discrepancy could be due to other factors governing the activity of the RGD adhesion domains. Indeed, it is known now that the simple presence of RGD sequences on the surface of a substratum is not sufficient to assure cell-adhesive properties and generate focal adhesions [22,69]. The RGD tripeptide is a ubiquitous ligand, being recognized by all five αV integrins, two β1 integrins and by the αIIbβ3 integrin [70]. However, a host of factors have critical roles in an effective recognition mechanism [69,71,72,73,74,75,76], including: surface density of RGD units; design of the RGD-containing precursor peptide, *i.e*., the nature of flanking amino acids; presence of adequate ‘spacers’ able to assist with the presentation of the exposed loop that contains RGD; nanoscale spatial distribution of the RGD sequences (clustering and interspacing). No assays to investigate any of these factors in the case of silk fibroin substrata have been carried out so far. 

## 5. Conclusions

The presence of the RGD peptide ligand is not sufficient to promote a statistically significant enhancement of cell attachment and growth on substrata of *Bombyx mori* silk fibroin, when the study model system is human corneal limbal epithelial (HLE) cells. While a trend towards an improvement in the attachment of cells can be noted, other factors may affect the adhesion ligand efficacy, warranting further investigations.

## References

[1] Minoura N., Tsukada M., Nagura M. (1990). Physico-chemical properties of silk fibroin membrane as a biomaterial. Biomaterials.

[2] Minoura N., Aiba S., Higuchi M., Gotoh Y., Tsukada M., Imai Y. (1995). Attachment and growth of fibroblast cells on silk fibroin. Biochem. Biophys. Res. Commun..

[3] Minoura N., Aiba S., Gotoh Y., Tsukada M., Imai Y. (1995). Attachment and growth of cultured fibroblast cells on silk protein matrices. J. Biomed. Mater. Res..

[4] Altman G.H., Diaz F., Jakuba C., Calabro T., Horan R.L., Chen J., Lu H., Richmond J., Kaplan D.L. (2003). Silk-based biomaterials. Biomaterials.

[5] Wang Y., Kim H.-J., Vunjak-Novakovic G., Kaplan D.L. (2006). Stem cell-based tissue engineering with silk biomaterials. Biomaterials.

[6] Vepari C., Kaplan D.L. (2007). Silk as a biomaterial. Prog. Polym. Sci..

[7] Hakimi O., Knight D.P., Vollrath F., Vadgama P. (2007). Spider and mulberry silkworm silks as compatible biomaterials. Composites B.

[8] Wang X., Cebe P., Kaplan D.L., Lutz S., Bornscheuer U.T. (2009). Silk Proteins—Biomaterials and bioengineering. Protein Engineering Handbook.

[9] Murphy A.R., Kaplan D.L. (2009). Biomedical applications of chemically-modified silk fibroin. J. Mater. Chem..

[10] Hardy J.G., Scheibel T.R. (2010). Composite materials based on silk proteins. Prog. Polym. Sci..

[11] Numata K., Kaplan D.L. (2010). Silk-based delivery systems of bioactive molecules. Adv. Drug Deliv. Rev..

[12] Pritchard E.M., Kaplan D.L. (2011). Silk fibroin biomaterials for controlled release drug delivery. Expert Opin. Drug Deliv..

[13] Wenk E., Merkle H.P., Meinel L. (2011). Silk fibroin as a vehicle for drug delivery applications. J. Control. Rel..

[14] Zhang Y.-Q. (2002). Applications of natural silk protein sericin in biomaterials. Biotechnol. Adv..

[15] Kundu S.C., Dash B.C., Dash R., Kaplan D.L. (2008). Natural protective glue protein, sericin bioengineered by silkworms: Potential for biomedical and biotechnological applications. Prog. Polym. Sci..

[16] Armato U., Dal Prà I., Chiarini A., Freddi G. (2011). Will silk fibroin nanofiber scaffolds ever hold a useful place in translational regenerative medicine?. Int. J. Burn Trauma.

[17] Chirila T.V., Barnard Z., Zainuddin, Harkin D.G., Schwab I.R., Hirst L.W. (2008). *Bombyx mori* silk fibroin membranes as potential substrata for epithelial constructs used in the management of ocular surface disorders. Tissue Eng. Part A.

[18] Ghassemifar R., Redmond S., Zainuddin, Chirila T.V. (2010). Advancing towards a tissue-engineered tympanic membrane: Silk fibroin as a substratum for growing human eardrum keratinocytes. J. Biomater. Appl..

[19] Madden P.W., Lai J.N.X., George K.A., Giovenco T., Harkin D.G., Chirila T.V. (2011). Human corneal endothelial cell growth on a silk fibroin membrane. Biomaterials.

[20] Bray L.J., George K.A., Hutmacher D.W., Chirila T.V., Harkin D.G. (2012). A dual-layer silk fibroin scaffold for reconstructing the human corneal limbus. Biomaterials.

[21] Shadforth A.M.A., George K.A., Kwan A.S., Chirila T.V., Harkin D.G. (2012). The cultivation of human retinal pigment epithelial cells on *Bombyx mori* silk fibroin. Biomaterials.

[22] Leal-Egaña A., Scheibel T. (2012). Interactions of cells with silk surfaces. J. Mater. Chem..

[23] Lotz B., Collona-Cesari F. (1979). The chemical structure and the crystalline structures of *Bombyx mori* silk fibroin. Biochimie.

[24] Mita K., Ichimura S., James T.C. (1994). Highly repetitive structure and its organization of the silk fibroin gene. J. Mol. Evol..

[25] Zhou C.-Z., Confalonieri F., Medina N., Zivanovic Y., Esnault C., Yang T., Jacquet M., Janin J., Duguet M., Perasso R. (2000). Fine organization of *Bombyx mori* fibroin heavy chain gene. Nucl. Acids Res..

[26] Yukuhiro K., Kanda T., Tamura T. (1997). Preferential codon usage and two types of repetitive motifs in the fibroin gene of the Chinese oak silkworm, *Antheraea pernyi*. Insect Mol. Biol..

[27] Sezutsu H., Yukuhiro K. (2000). Dynamic rearrangement within the *Antheraea pernyi* silk fibroin gene is associated with four types of repetitive units. J. Mol. Evol..

[28] Hwang J.-S., Lee J.-S., Goo T.-W., Yun E.-Y., Lee K.-S., Kim Y.-S., Jin B.-R., Lee S.-M., Kim K.-Y., Kang S.-W. (2001). Cloning of the fibroin gene from the oak silkworm, *Antheraea yamamai* and its complete sequence. Biotechnol. Lett..

[29] Zheng Z., Wei Y., Yan S., Li M. (2010). Preparation of regenerated *Antheraea yamamai* silk fibroin film and controlled-molecular conformation changes by aqueous ethanol treatment. J. Appl. Polym. Sci..

[30] Lv L., Wei Y., Wang J., Li M., Zhao H., Liu G., Lv Q. Preparation and Physical Properties of *Antheraea yamamai*/*Bombyx mori* Silk Fibroin Blending Film. Proceedings of the 4th International Conference on Biomedical Engineering and Informatics (BMEI).

[31] Acharya C., Ghosh S.K., Kundu S.C. (2009). Silk fibroin film from non-mulberry tropical tasar silkworms: A novel substrate for *in vitro* fibroblast culture. Acta Biomater..

[32] Datta A., Ghosh A.K., Kundu S.C. (2001). Differential expression of the fibroin gene in developmental stages of silkworm, *Antheraea mylitta* (Saturniidae). Comp. Biochem. Physiol. Part B.

[33] Gotoh Y., Tsukada M., Minoura N. (1998). Effect of the chemical modification of the arginyl residue in *Bombyx mori* silk fibroin on the attachment and growth of fibroblast cells. J. Biomed. Mater. Res..

[34] Yamada H., Igarashi Y., Takasu Y., Saito H., Tsubouchi K. (2004). Identification of fibroin-derived peptides enhancing the proliferation of cultured human skin fibroblasts. Biomaterials.

[35] Sofia S., McCarthy M.B., Gronowicz G., Kaplan D.L. (2001). Functionalized silk-based biomaterials for bone formation. J. Biomed. Mater. Res..

[36] Kardestuncer T., McCarthy M.B., Karageorgiou V., Kaplan D., Gronowicz G. (2006). RGD-tethered silk substrate stimulates the differentiation of human tendon cells. Clin. Orthop. Rel. Res..

[37] Kim J.W., Ki C.S., Park Y.H., Kim H.J., Um I.C. (2010). Effect of RGDS and KRSR peptides immobilized on silk fibroin nanofibrous mats for cell adhesion and proliferation. Macromol. Res..

[38] Yanagisawa S., Zhu Z., Kobayashi I., Uchino K., Tamada Y., Tamura T., Asakura T. (2007). Improving cell-adhesive properties of recombinant *Bombyx mori* silk by incorporation of collagen or fibronectin derived peptides produced by transgenic silkworms. Biomacromolecules.

[39] Morgan A.W., Roskov K.E., Lin-Gibson S., Kaplan D.L., Becker M.L., Simon C.G. (2008). Characterization and optimization of RGD-containing silk blends to support osteoblastic differentiation. Biomaterials.

[40] Yang M., Tanaka C., Yamauchi K., Ohgo K., Kurokawa M., Asakura T. (2008). Silklike materials constructed from sequences of *Bombyx mori* silk fibroin, fibronectin and elastin. J. Biomed. Mater. Res..

[41] Kambe Y., Yamamoto K., Kojima K., Tamada Y., Tomita N. (2010). Effects of RGDS sequence genetically interfused in the silk fibroin light chain protein on chondrocyte adhesion and cartilage synthesis. Biomaterials.

[42] Hu X., Wang X., Rnjak J., Weiss A.S., Kaplan D.L. (2010). Biomaterials derived from silk-tropoelastin protein systems. Biomaterials.

[43] Hu X., Park S.-H., Gil E.S., Xia X.-X., Weiss A.S., Kaplan D.L. (2011). The influence of elasticity and surface roughness on myogenic and osteogenic-differentiation of cells on silk-elastin biomaterials. Biomaterials.

[44] Gil E.S., Mandal B.B., Park S.-H., Marchant J.K., Omenetto F.G., Kaplan D.L. (2010). Helicoidal multi-lamellar features of RGD-functionalized silk biomaterials for corneal tissue engineering. Biomaterials.

[45] Huang Y., Ren J., Ren T., Gu S., Tan Q., Zhang L., Lv K., Pan K., Jiang X. (2010). Bone marrow stromal cells cultured on poly(lactide-co-glycolide)/nano-hydroxyapatite composites with chemical immobilization of Arg-Gly-Asp peptide and preliminary bone regeneration of mandibular defect thereof. J. Biomed. Mater. Res..

[46] Bray L.J., George K.A., Ainscough S.L., Hutmacher D.W., Chirila T.V., Harkin D.G. (2011). Human corneal epithelial equivalents constructed on *Bombyx mori* silk fibroin membranes. Biomaterials.

[47] Suzuki S., Bray L.J., Edwards G.A., Chirila T.V. (2013).

[48] Tretinnikov O.N., Tamada Y. (2001). Influence of casting temperature on the near-surface structure and wettability of cast silk fibroin films. Langmuir.

[49] Murphy A.R., St. John P., Kaplan D.L. (2008). Modification of silk fibroin using diazonium coupling chemistry and the effects on hMSC proliferation and differentiation. Biomaterials.

[50] Vepari C., Matheson D., Drummy L., Naik R., Kaplan D.L. (2010). Surface modification of silk fibroin with poly(ethylene glycol) for antiadhesion and antithrombotic applications. J. Biomed. Mater. Res..

[51] Sampaio S., Miranda T.M.R., Santos J.G., Soares G.M.B. (2011). Preparation of silk fibroin-poly(ethylene glycol) conjugate films through click chemistry. Polym. Int..

[52] Das S., Pati D., Tiwari N., Nisal A., Sen Gupta S. (2012). Synthesis of silk fibroin-glycopolypeptide conjugates and their recognition with lectin. Biomacromolecules.

[53] Horbett T.A., Klumb L.A., Brash J.L., Wojciechowski P.W. (1996). Cell culturing: Surface aspects and considerations. Interfacial Phenomena and Bioproducts.

[54] De Giglio E., Sabbatini L., Colucci S., Zambonin G. (2000). Synthesis, analytical characterization, and osteoblast adhesion properties on RGD-grafted polypyrrole coatings on titanium substrates. J. Biomater. Sci. Polym. Ed..

[55] Chirila T.V., Minamisawa T., Keen I., Shiba K. (2009). Effect of motif-programmed artificial proteins on the calcium uptake in a synthetic hydrogel. Macromol. Biosci..

[56] Merrett K., Griffith C.M., Deslandes Y., Pleizier G., Sheardown H. (2001). Adhesion of corneal epithelial cells to cell adhesion peptide modified pHEMA surfaces. J. Biomater. Sci. Polym. Ed..

[57] Kweon H., Park Y.H. (2001). Dissolution and characterization of regenerated *Antheraea pernyi* silk fibroin. J. Appl. Polym. Sci..

[58] Tsukada M., Freddi G., Gotoh Y., Kasai N. (1994). Physical and chemical properties of tussah silk fibroin films. J. Polym. Sci. Part B.

[59] Li M., Tao W., Lou S., Kuga S. (2003). Compliant film of regenerated *Antheraea pernyi* silk fibroin by chemical crosslinking. Int. J. Biol. Macromol..

[60] Zuo B., Liu L., Zhang F. (2009). Structure and properties of regenerated *Antheraea pernyi* silk fibroin filaments. J. Appl. Polym. Sci..

[61] George K., Bray L.J. (2012).

[62] Mandal B.B., Kundu S.C. (2008). A novel method for dissolution and stabilization of non-mulberry silk gland protein fibroin using anionic surfactant sodium dodecyl sulfate. Biotechnol. Bioeng..

[63] Mandal B.B., Kundu S.C. (2008). Non-bioengineered silk fibroin protein 3D scaffolds for potential biotechnological and tissue engineering applications. Macromol. Biosci..

[64] Mandal B.B., Das S., Choudhury K., Kundu S.C. (2010). Implication of silk film RGD availability and surface roughness on cytoskeletal organization and proliferation of primary rat bone marrow cells. Tissue Eng. Part A.

[65] Patra C., Talukdar S., Novoyatleva T., Velagala S.R., Mühlfeld C., Kundu B., Kundu S.C., Engel F.B. (2012). Silk protein fibroin from *Antheraea mylitta* for cardiac tissue engineering. Biomaterials.

[66] Wu X.F. (2009). The Study of Regenerated *Antheraea Pernyi*/*Bombyx Mori* Silk Fibroin Blend Porous Materials. M.S. Thesis.

[67] Qu J., Xin L., Xu X., Zhang F., Zuo B., Zhang H., Lim C.T., Goh J.C.H. (2010). Tussah Silk Fibroin Excels Silk Fibroin from the Domesticated Silkworm in Supporting the Development of Neurons. IFMBE Proceedings (The 6th World Congress of Biomechanics WCB 2010).

[68] Hakimi O., Gheysens T., Vollrath F., Grahn M.F., Knight D.P., Vadgama P. (2010). Modulation of cell growth on exposure to silkworm and spider silk fibers. J. Biomed. Mater. Res..

[69] Hersel U., Dahmen C., Kessler H. (2003). RGD modified polymers: Biomaterials for stimulated cell adhesion and beyond. Biomaterials.

[70] Humphries J.D., Byron A., Humphries M.J. (2006). Integrin ligands at a glance. J. Cell Sci..

[71] Massia S.P., Hubbell J.A. (1990). Covalent surface immobilization of Arg-Gly-Asp- and Tyr-Ile-Gly-Ser-Arg-containing peptides to obtain well-defined cell-adhesive substrates. Anal. Biochem..

[72] Massia S.P., Hubbell J.A. (1991). Human endothelial cell interactions with surface-coupled adhesion peptides on a nonadhesive glass substrate and two polymeric biomaterials. J. Biomed. Mater. Res..

[73] Massia S.P., Hubbell J.A. (1991). An RGD spacing of 440 nm is sufficient for integrin α_v_β_3_-mediated fibroblast spreading and 140 nm for focal contact and stress fiber formation. J. Cell Biol..

[74] Hern D.L., Hubbell J.A. (1998). Incorporation of adhesion peptides into nonadhesive hydrogels useful for tissue resurfacing. J. Biomed. Mater. Res..

[75] Elbert D.L., Hubbell J.A. (2001). Conjugate addition reactions combined with free-radical cross-linking for the design of materials for tissue engineering. Biomaterials.

[76] Huang J., Gräter S.V., Corbellini F., Rinck S., Bock E., Kemkemer R., Kessler H., Ding J., Spatz J.P. (2009). Impact of order and disorder in RGD nanopatterns on cell adhesion. Nano Lett..

